# Supplementary material for: No Remdesivir Resistance Observed in the Phase 3 Severe and Moderate COVID-19 SIMPLE Trials
Source: Viruses. 2024 Mar 31;16(4):546. doi: 10.3390/v16040546 (PMC11053423; doi:10.3390/v16040546)
Supplement: Supplementary file 1 [file viruses-16-00546-s001.zip › viruses-2924704-supplementary.pdf]

Table S1. Sequence data obtained per study site.

Study Site	Site Number	No. of Samples with Attempted Sequencing	No. of Samples with Successful Sequencing
University Hospital rechts der Isar Technical University of Munich, Germany	9030	93	51
Baylor University Medical Center, USA	14759, 17914	63	19
Academische Ziekenhuis Leiden, Netherlands	17712	41	23
Hospital Universitari Vall d'Hebron, Spain	17860	38	34
Klinik für Infektionskrankheiten und Spitalhygiene, Switzerland	15128	28	18
UH Clinical Research Center/University Hospital Case Western, USA	17719	17	16
Icahn School of Medicine at Mount Sinai, USA	18408, 18409, 18410	6	6
